# Allergic Bronchopulmonary Aspergillosis–A Luminal Hypereosinophilic Disease With Extracellular Trap Cell Death

**DOI:** 10.3389/fimmu.2018.02346

**Published:** 2018-10-11

**Authors:** Shigeharu Ueki, Akira Hebisawa, Masashi Kitani, Koichiro Asano, Josiane S. Neves

**Affiliations:** ^1^Department of General Internal Medicine and Clinical Laboratory Medicine, Akita University Graduate School of Medicine, Akita, Japan; ^2^Clinical Research Center and Pathology Division, National Hospital Organization Tokyo National Hospital, Tokyo, Japan; ^3^Division of Pulmonary Medicine, Department of Medicine, Tokai University School of Medicine, Kanagawa, Japan; ^4^Institute of Biomedical Sciences, Federal University of Rio de Janeiro, Rio de Janeiro, Brazil

**Keywords:** eosinophil, extracellular traps, extracellular trap cell death, allergic bronchopulmonary aspergillosis, inflammation, mucus plugs, ETosis, NETosis

## Abstract

Allergic bronchopulmonary aspergillosis (ABPA) is characterized by an early allergic response and late-phase lung injury in response to repeated exposure to *Aspergillus* antigens, as a consequence of persistent fungal colonization of the airways. Here, we summarize the clinical and pathological features of ABPA, focusing on thick mucus plugging, a key observation in ABPA. Recent findings have indicated that luminal eosinophils undergo cytolytic extracellular trap cell death (ETosis) and release filamentous chromatin fibers (extracellular traps, ETs) by direct interaction with *Aspergillus fumigatus*. Production of ETs is considered to be an innate immune response against non-phagocytable pathogens using a “trap and kill” mechanism, although eosinophil ETs do not promote *A. fumigatus* damage or killing. Compared with neutrophils, eosinophil ETs are composed of stable and condensed chromatin fibers and thus might contribute to the higher viscosity of eosinophilic mucus. The major fate of massively accumulated eosinophils in the airways is ETosis, which potentially induces the release of toxic granule proteins and damage-associated molecular patterns, epithelial damage, and further decreases mucus clearance. This new perspective on ABPA as a luminal hypereosinophilic disease with ETosis/ETs could provide a better understanding of airway mucus plugging and contribute to future therapeutic strategies for this challenging disease.

## Introduction

Allergic bronchopulmonary aspergillosis (ABPA) is a disease entity first proposed by Hinson and colleagues as bronchopulmonary aspergillosis in 1952 (1). ABPA develops mainly in adolescent and adult patients with asthma or cystic fibrosis. It has been estimated that 2.5% (0.7–3.5%) of adult patients with asthma suffer from ABPA (2). Clinically, it is characterized by peripheral blood eosinophilia, increased levels of serum IgE, an immediate skin reaction and/or specific IgE/IgG antibodies to *Aspergillus fumigatus* due to type I and III hypersensitivity reactions, and radiographic findings including pulmonary opacities, central bronchiectasis, and mucus plugs (3–5). Systemic corticosteroids and/or anti-fungal drugs are effective, although approximately half of ABPA patients experience relapse (6, 7).

Besides blood eosinophilia, a massive accumulation of eosinophils and clustering of these immune cells in the bronchial lumen, resulting in bronchial impaction, are hallmarks of ABPA. Clinically, this has been defined by different terms depending on the context, such as “allergic mucin,” “high attenuation mucus,” or “allergic mucus plugs.” Considerable evidence has indicated the close association between the sputum eosinophil count and/or the eosinophil granule protein concentration and asthma severity (8, 9), although much less attention has been paid to luminal eosinophils (and their lytic components) in ABPA. This may be simply because of the lack of an explicit relationship and/or the difficulties in measuring protein concentrations due to the inspissated bronchial secretions. In this review, we discuss the clinical features of ABPA, focusing on new insights into the fate of eosinophils and their “cell debris” in the airways.

## Fungus in the airways

Germination and saprophytic growth of fungi in the mucus are interesting and unique features of ABPA. *Aspergillus fumigatus* is the major causative fungus of ABPA, but other *Aspergillus* spp., such as *A. flavus, A. niger*, and *A. oryzae*, can also cause ABPA, although less frequently. *Schizophyllum commune*, a filamentous basidiomycete commonly found on the rotten wood of trees, can cause similar pathology and a condition known as allergic bronchopulmonary mycosis (ABPM) (7, 10–12). Because fungal hyphae are immunologically active expressing and releasing various proteases and pathogen-associated molecular patterns (13–15), repeated exposures to airborne fragments of fungal hyphae, either dead or alive, can cause asthma and hypersensitivity pneumonitis. For the development of ABPM, by contrast, inhalation of viable fungi as conidia, not their hyphae fragments, and their germination in the lower airways is essential. *A. fumigatus* has advantages for the development of ABPA/ABPM over other fungi because of the small size of its conidia (3–6 μm) and thermophilicity (16). The conidia of *S. commune* are also small (3–4 μm), and prefer a relatively high temperature (30–35°C) to germinate (17). *A. fumigatus* conidia are also known for their high dispersibility due to their remarkable hydrophobicity (18).

Unlike fungal infections, germinated hyphae cannot penetrate the lung tissues in the presence of a normal immune system and bronchial structure. Therefore, *A. fumigatus* remains in the mucus plugs of the bronchi. Compared with other fungi, *A. fumigatus* has another advantage in this process in that it induces the *Muc5ac* gene, one of the mucin genes, and mucus production in bronchial epithelial cells (19). Induction of *Muc5ac* gene expression is dependent on the high serine protease activity of *A. fumigatus*, which activates membrane-bound TNFα-converting enzyme and TGF-α, stimulating epidermal growth factor receptors. Compared with *A. fumigatus*, other fungi, such as *Penicillium* and *Alternaria*, and other *Aspergillus* spp., such as *A. flavus* and *A. niger* have lower serine protease activity (19, 20).

## Radiographic features of ABPA

In the first description of ABPA, Hinson and colleagues advocated that the features of ABPA included (i) wheezing and blood eosinophilia, (ii) repetitive infiltrations visible on chest X-ray, and (iii) “allergic” (eosinophilic) mucus plugs with fungal hyphae (AMwF) (1). In 1967, Scadding (21) reported the presence of central bronchiectasis by bronchography, with the initial mucus-filled bronchi being less apparent but bronchial ectasis remaining. The diagnostic criteria for ABPA in the pre-computerized tomography era, proposed by Rosenberg and Patterson (22), included pulmonary opacities and central bronchiectasis.

Currently, the radiographic features of ABPA include bronchiectasis, mucoid impaction, pulmonary opacities, mosaic attenuation, centrilobular nodules, tree-in-bud opacities, and pleuropulmonary fibrosis (3). Pulmonary opacities, which are usually transient, are frequently observed during the course of the disease, with 89% of ABPA cases with bronchiectasis demonstrating pulmonary opacities/ground grass attenuation in a nationwide survey in Japan (7); however, this is not classed as a specific feature of this disease. Central bronchiectasis with peripheral tapering of bronchi has been considered as a relatively specific finding for ABPA, but bronchiectasis can extend to the peripheral bronchi in 33–63% of cases (23, 24). Mucus plugs in the bronchi are common in ABPA, and may present as high attenuation mucus (HAM) with a CT density higher than the values of paraspinal skeletal muscle (25) or 70 Hounsfield units (26). Magnetic resonance imaging of HAM showed hypodense lesions in T1- and T2-weighed images, suggesting that the mucus is desiccated or inspissated (27). HAM can be observed in more than half of the cases with mucus plugs due to ABPA (7, 23), and is more specific for this disease. The presence of HAM also correlates with a higher number of eosinophils in the peripheral blood and greater susceptibility to disease relapse (6, 28).

## Clinicopathological features of ABPA

Regarding the pathological features of ABPA, Katzenstein and colleagues emphasized the presence of AMwF, bronchocentric granulomatosis with tissue eosinophilia (BrCG-eo), eosinophilic bronchiolitis, and eosinophilic pneumonia (29). In 1988, Bosken and coworkers indicated, based on the investigation of surgically resected specimens, that mucoid impaction of bronchi with allergic mucin or BrCG-eo, together with fungal hyphae detected in the lesion, were sufficient for the diagnosis of ABPA/ABPM (30). However, the diagnostic criteria proposed by Rosenberg and Patterson (22) excluded pathological findings; instead, clinical, radiological, and laboratory findings, especially allergy tests, were included.

AMwF was detected in the biopsy samples by bronchoscopy, expectorated airway secretions, and the resected lungs. AMwF has been described by pathologists as follows (31, 32). “*AMwF could be found in any bronchi of any lobes, including lobar and segmental bronchi and 3rd−5th bifurcated small bronchi. AMwF was typically white-yellowish, dark-yellowish, or yellow-greenish in color, and sticky with elastic soft or elastic hard in consistency. The shape ranged from branching and clubbing to lumpy. The H&E stained section of the AMwF demonstrated concentric multiple layers consisting of eosinophils, Charcot-Leyden crystals, and fibrin (32, 33). The eosinophils might be viable, necrobiotic (with pyknotic nuclei and nuclear dusts) or necrotic. The latter two types formed inspissated eosinophil aggregates with indistinct cell margins and occasionally exhibited many fissures likely due to dehydration inside of AMwF, demonstrating ‘fir-tree structure’ (33). Fungi were usually found in the clusters of eosinophils as fragmented hyphae or branching septate hyphae, and occasionally in the mucus surrounding the eosinophil clusters. While fungi floats in the air as unicellular spores, they reside in the AMwF as multicellular hyphae, suggesting the proliferation of fungi*.”

Central bronchiectasis and peripheral lung lesions in ABPM were suggested as secondary pathological changes after the formation of AMwF. BrCG-eo, eosinophilic bronchiolitis, and eosinophilic pneumonia were found to be in the airways peripheral to the AMwF plugs, previously described as follows (32). “*The investigation of the resected lungs of ABPM patients revealed the airway walls plugged by AMwF were invaded with eosinophils and small round cells including plasma cells and were ulcerated, resulting in eosinophilic infiltration into the allergic mucin. The intense inflammation in the bronchial walls destructed and dissipated airway structures i.e., elastic fibers and smooth muscles, and even bronchial cartilages. Central bronchiectasis could be caused by the vulnerability of diseased bronchial walls and by expanding AMwF. In the exudate from BrCG-eo and eosinophilic bronchiolitis, inspissated eosinophil aggregates, fir-tree structures and fungi were detected, similar to the AMwF. However, the inspissated eosinophil aggregates and fir-tree structures were fragmented and the number of fungi was smaller than that in the central lesions of AMwF (30–33). These findings suggested that the peripheral lung lesions might be formed by the bronchogenic spread of AMwF to the peripheral lung*.” Thus, careful morphological analysis has indicated the AMwF is an initial and crucial step in the development of ABPA.

## Eosinophil extracellular trap cell death (ETosis) and extracellular traps

Eosinophils are bone marrow-derived, short-lived, non-dividing granulocytes that have been implicated as integral components of allergic airway inflammation. Eosinophils contain ~200 granules per cell (34), which are preformed stores of the specific granule proteins (35). The highly cationic granule proteins possess cytotoxicity by disrupting the integrity of lipid bilayers, exerting neurotoxic properties, RNase activities, and participating in the generation of reactive oxidants and radical species (36, 37). Since eosinophil degranulation does not occur in the circulation (38), it is critical to understand the actual mode of degranulation and cell fate in the airways. Our recent studies indicated eosinophil cell-death mediated degranulation, i.e., eosinophil ETosis, which might play an important role in the generation of mucus plugs (39, 40).

In 2004, Brinkmman and colleagues provided the first evidence that neutrophils release filamentous chromatin structures, i.e., neutrophil extracellular traps (NETs) (41). A subsequent study revealed that the novel cell death process mediates the release of NETs and was designated NETosis (42). NETosis has unique features that are different from apoptosis and necrosis. Unlike apoptosis, nuclear DNA fragmentation is spared during the process of NETosis. Histone citrullination induces chromatin decondensation and granules are intracellularly disrupted before plasma membrane disintegration, thereby NETs are associated with various antimicrobial molecules (including histones, elastase, myeloperoxidase, cathepsin, and lactoferrin). Once NETs are released, they immobilize and potentially kill pathogens (41, 43, 44). Based on this, roles for these structures in preventing microbial spread and in creating an antimicrobial environment have been postulated.

Since chromatin traps are cytolytically released by other leukocytes, such as macrophages (45) and mast cells (46, 47), “ETosis” has been proposed as a similar cell death pathway (48). Extracellular trap (ET) formation is now considered to be a common mechanism of the innate immune system in vertebrates (49). A recent report showed that even invertebrate cells are capable of ETosis, indicating an ancient and evolutionary conserved mechanism (50). Eosinophil ETosis (EETosis) was first reported as a mechanism of “cytolysis” (also called lytic degranulation, necrosis, primary lysis, and eosinophil lysis) that has been observed (but often overlooked) in eosinophilic inflammatory diseases (35). This finding indicates that eosinophil cytolysis does not represent a process of passive/accidental necrosis, rather eosinophils are actively selecting their death program at the inflammatory site. The process of EETosis is akin to that of neutrophils but notably differs in that intact granules are released extracellularly (35, 51–53).

It is well-established that the process of leukocyte activation and ET release plays a role in health and disease, including innate immunity, autoimmunity, metabolic disorders, malignancies, and coagulation (44, 46, 54–57). Excess production of ETs could be pathogenic (49); for instance, large amounts of NETs contribute to the thickness of airway fluids from patients with cystic fibrosis (58). Inhalation of recombinant human DNase improved lung function in cystic fibrosis patients, indicating the pathogenicity of NETs (58–60). Similar to NETs, EETs provide a sticky scaffold for secretions, a fact confirmed by the decrease in viscosity following disruption of EET polymers with DNase (61). Recently, we have shown abundant EETs associated with clusters of cell-free extracellular granules in the bronchial mucus plugs of ABPA patients (39, 40). We also observed a drastic decrease in EETs in the bronchoscopically-obtained secretions from an ABPA patient after corticosteroid treatment, which correlated with clinical improvements (40).

It is noteworthy that NETs and EETs have different attributes in terms of stability and structure. The protein content of ETs plays an important role in its mechanical properties (62). Compared with neutrophils, eosinophils contain far less proteases, and thus eosinophil chromatin is spared from endogenous protease digestion (61, 63). Neutrophil elastase promotes the chromatin decondensation of NETs by proteolytic processing of histones (64). *In vitro*, NETs deteriorated within 24 h, whereas EETs were stable for at least 7 days (61). Electron microscopy showed that released NETs consisted of 5–10-nm smooth stretches (composed of stacked cylindrical nucleosomes) and 25–50-nm globular domains (41, 65), although EETs consisted mostly of chromatin fibers with diameters of 25–35 nm in conjunction with larger fibers (35). In terms of innate immune responses, staunch fibers might offer an advantage by immobilizing and hampering the progression of large parasites (66), and may also pathologically contribute to the highly viscous nature of eosinophil-dominant airway secretions, as clinically observed in ABPA, but also in other diseases, such as eosinophil chronic rhinosinusitis (51, 61), eosinophilic otitis media (63, 67), and severe asthma (40, 68).

There is evidence *in vitro* that human eosinophils rapidly activate in response to various stimuli by releasing mitochondrial DNA via a non-cytolytic mechanism (69, 70). The mitochondrial DNA would be present in mucus plugs since EETosis liberates total cellular contents through plasma membrane disintegration. However, mitochondrial DNA, lacking the histone and nucleosome structure, is only 16 kbp in size, constituting <1% of total cellular DNA (e.g., genomic DNA has 3.2 billion bp, which equates to 2 m in length/cell) (71). In addition, human eosinophils contain a small number of mitochondria (~30/cell) (72). Therefore, nuclear-derived chromatin fibers are a major component contributing to viscosity (35), as evidenced by positive staining of airway EETs with specific antibodies against histones/citrullinated histones (39, 61).

## *A. fumigatus* induced eosinophil ETosis

The immunopathological mechanisms that underlie the molecular interactions between *A. fumigatus* and eosinophils are also of interest (see review (73)). Our *in vitro* studies have shown that ETs are released by lytic human eosinophils in direct response to *A. fumigatus* (39). When human eosinophils were co-cultured with *A. fumigatus*, EETs were released in a time and ratio (fungus: cell) dependent manner (39).

Unlike necrosis, ETosis is an active form of cell death and is frequently accompanied by the production of reactive oxygen species (ROS) (35, 41, 43, 52, 69, 70, 74–76); however, we observed that human eosinophils release EETs *in vitro* in response to *A. fumigatus* in a ROS-independent manner (39). Regarding *Aspergillus* specifically, neutrophils are described to respond to different morphotypes of this fungus by releasing NETs in a ROS-dependent process (77–79). However, the ROS-independent release of NETs was observed when stimulated by bacteria (80), parasites (81), fungi (*Candida* and *Paracoccidioides brasiliensis* conidia) (82, 83), and other stimuli (84–86). The participation of ROS in the process of ETosis might be essentially dependent on the stimuli, time point, or microorganisms examined.

Mechanistically, *A. fumigatus* induced EETosis *in vitro* is a process dependent on the Syk tyrosine kinase pathway (known to mediate cell signaling via different classes of receptors involved in fungus recognition) and adhesion molecule CD11b β-integrin (which recognizes β-glucan, a molecular pattern commonly found in the cell wall of fungi) (39). Consistent with these findings, CD11b has been described to play an important role in eosinophil interactions with the fungus *Alternaria alternata* (87). Previous studies with eosinophils and *A. alternata* suggest a critical role for the CD11b I domain in eosinophil activation and degranulation, but not for the lectin domain, which recognizes the β-glucan (87). In neutrophils, both the C-type lectin receptor dectin-1 and the β-integrin CD11b/CD18 (CR3) have been implicated in the recognition of β-glucans on the cell surface of *A. fumigatus* (79, 88, 89). However, we did not detect the expression of dectin-1 protein in human blood eosinophils (39).

Airway eosinophils display enhanced responses to a variety of ligands and become further activated to degranulate (90, 91). Human eosinophils undergo ETosis in response to immobilized IgG or IgA (used as *in vitro* defined models of immunoglobulin-coated pathogens including parasites), calcium ionophore, IL-5/GM-CSF with platelet activating factor, or phorbol myristate acetate to liberate ETs (35, 68, 92). In addition to the direct response to *A. fumigatus*, it is possible that locally produced immunoglobulins, cytokines, and other mediators could activate eosinophils to induce EETosis. Indeed, a recent study indicated that IgG-type autoantibodies were present in the airways of patients with severe eosinophilic asthma, potentially induced by the release of EETs (68). It is also noteworthy that biological molecules associated with the fungal cell wall, such as chitin and β-glucan from *Aspergillus* species, induce eosinophilic Th2 inflammation in mouse lungs (93–95).

## Luminal eosinophils and ETosis matter

EETs exhibited intimate contact with *A. fumigatus* conidia and with cell-free extracellular granules, indicating that EETs provide an adhesive surface for organelle and microorganism entrapment (39). The highly hydrophobic surface of *A. fumigatus* might be easily entrapped by EETs, since EETs ensnare microorganisms mainly via hydrophobic interactions (39, 61). It has been reported that NETs may efficiently kill microbial pathogens (96–99); however, several fungi may resist the antimicrobial effect of NETs (79, 83, 100, 101).

Interestingly, our study revealed that human EETs did not promote the damage or killing of *A. fumigatus* (39). Eosinophils exhibit killing activity against different pathogens both *in vitro* and in experimental models (reviewed in 102). *In vitro*, Yoon and colleagues demonstrated that human eosinophils release their cytotoxic granule proteins in response to *A. alternata* and kill the fungus in a contact-dependent manner (87). Mouse bone marrow-derived eosinophils exhibited killing activity against *A. fumigatus in vitro*, which did not require cell contact (103). By contrast, using a Th2-dominant murine model of chronic invasive aspergilosis, eosinophil-deficient mice showed decreased morbidity and improved clearance of *A. fumigatus* (94). Intriguingly, studies based on mouse experimental models revealed that Th2-type immunity to chronic fungi exposition is generally accompanied by detrimental allergic inflammation, including tissue eosinophilia, goblet cell hyperplasia, and airway remodeling, but no signs of hyphae or invasive fungal growth were reported at any time after conidia challenge (104). Thus, the development of an animal model showing fungal colonization of the airways similar to that observed in ABPA pathology is still lacking. It remains unclear whether these discrepancies are due to the differences between mouse and human eosinophils (105) and/or experimental settings. Nevertheless, *A. fumigatus* resistance to EET killing activity might be an important feature of ABPA pathogenesis, possibly explaining the previously suggested “innate immunological defect” in this disease (3).

The rheological properties of mucus and the mucociliary transport system function as a self-cleaning mechanism for the respiratory tract (106). However, difficult-to-remove eosinophilic airway secretions are associated with disease severity (107, 108). As described above, the lower protease content and stable chromatin traps of eosinophils contribute to airway mucus viscosity. Most recently, Dunican and coworkers indicated that oxidants generated by eosinophil granule protein oxidize cysteine thiol groups to stiffen airway mucus (109).

**Figure 1:**
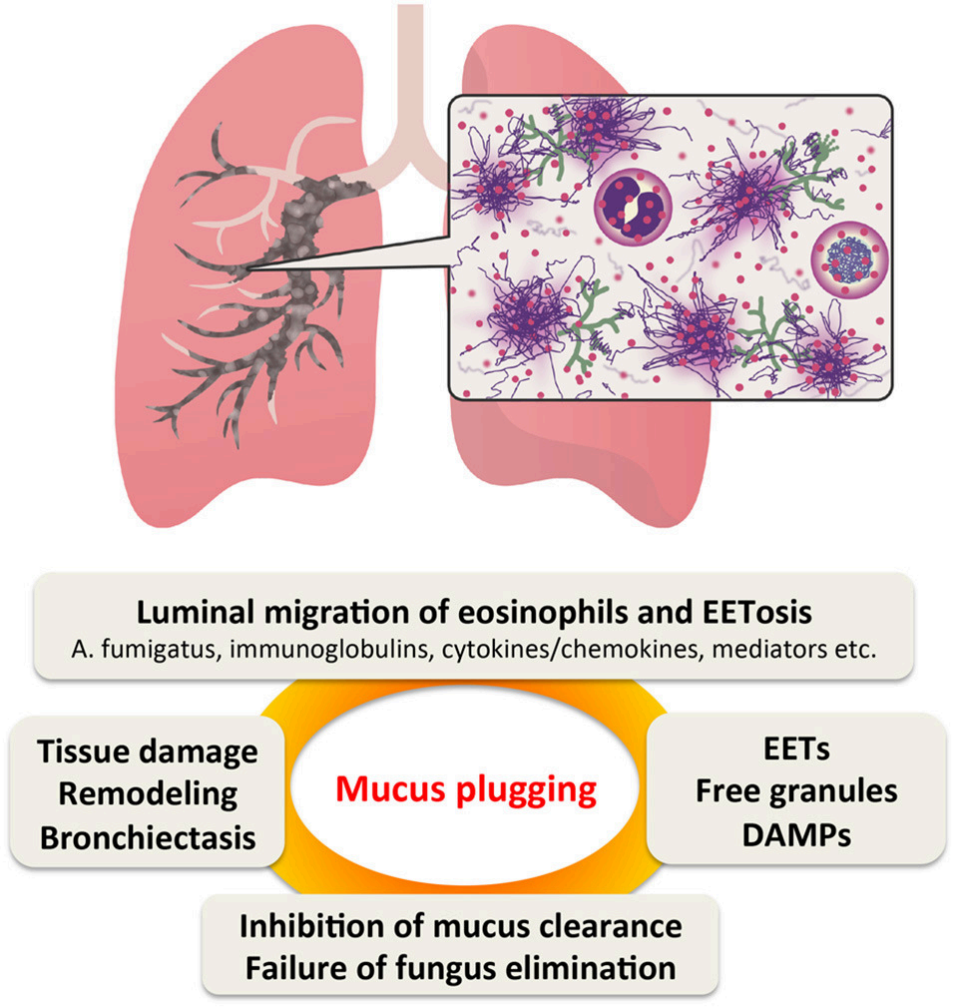
Proposed perpetuating cycle of mucus plugging caused by eosinophil ETosis (EETosis). In Th2-type airway inflammation, eosinophils are the major effector cells. They are supplied from the blood and massively accumulate in the bronchial lumen, where they are highly activated by fungi and other stimulants. EETosis-mediated cytolysis releases their total cellular contents including eosinophil extracellular traps (EETs), which contributes to the higher viscosity of eosinophilic mucus. Persistent fungal colonization of the airways and inhibition of “cytotoxic” mucus clearance further contributes to tissue damage, resulting in bronchiectasis. DMAPs, damage-associated molecular patterns.

We propose that EETosis contributes significantly to the fate of luminal eosinophils and plays a critical role in the pathogenesis of ABPA (Figure 1). Under Th2 conditions, blood eosinophils actively accumulate in the airway tissue. Eosinophils are usually eliminated by migration into the airway lumen followed by mucociliary clearance (110). Unlike apoptosis that produces fragmented DNA, luminal eosinophils undergo ETosis by direct interaction with fungi and/or local stimuli to release a sticky chromatin structure. EETs contribute to the increased viscosity of mucus rather than the direct elimination of fungi. Eosinophil cytolysis also releases intact granules and a wide range of nuclear and cytosolic damage-associated molecular patterns (92). Free granules may act as a reservoir of toxic granule proteins and secretion-competent extracellular organelles (35, 111). Perpetuating epithelial damage may inhibit the effectiveness of ciliary beating, thereby decreasing mucus transport and also resulting in the release of alamins and further eosinophilic inflammation. Thus, EETosis might promote a perpetuating cycle of thickening secretions (51). However, this new perspective requires further evaluation.

## Concluding remarks

As reviewed by Persson and Uller (112), cytolysis has often been overlooked by researchers, although several clinical studies illustrated that cytolysis accounted for a major proportion of bronchial eosinophils in severe and lethal asthma (112). Because apoptosis is a rare event among tissue-migrated eosinophils, ETosis-mediated cytolysis and luminal entry are likely to be the major fates of these cells. Indeed, Charcot–Leyden crystals, often observed in mucus plugs, were generated in association with the EETosis process (53). Considering the clinical features of ABPA, it is conceivable that increased eosinophil turnover, rather than prolonged lifespan, might be a major cause of luminal eosinophilia. It should be recognized that EETosis-mediated cytolysis and EETs are potentially important therapeutic targets in ABPA, as well as in other eosinophilic inflammatory diseases.

## Author contributions

SU wrote and finalize the manuscript. AH and MK contributed pathological part; KA contributed clinical part. JN wrote cell biology part.

### Conflict of interest statement

The authors declare that the research was conducted in the absence of any commercial or financial relationships that could be construed as a potential conflict of interest.

## Abbreviations

ABPA: allergic bronchopulmonary aspergillosis
ABPM: allergic bronchopulmonary mycosis
AMwF: allergic mucus plugs with fungal hyphae
BrCG-eo: bronchocentric granulomatosis with tissue eosinophilia
ET: extracellular trap
EET: eosinophil extracellular trap
ETosis: extracellular trap cell death
EETosis: eosinophil extracellular trap cell death
HAM: high attenuation mucus
NET: neutrophil extracellular trap
NETosis: neutrophil extracellular trap cell death
ROS: reactive oxygen species
